# Design of Sparse FIR Decision Feedback Equalizers in MIMO Systems Using Hybrid *l*_1_/*l*_2_ Norm Minimization and the OMP Algorithm

**DOI:** 10.3390/s18061860

**Published:** 2018-06-06

**Authors:** Lihong Yu, Jiaxiang Zhao, Wei Xu, Haiyuan Liu

**Affiliations:** 1 College of Computer and Control Engineering, Nankai University, Tianjin 300350, China; yulihongfly@mail.nankai.edu.cn; 2 Tianjin Key Laboratory of Optoelectronic Sensor and Sensing Network Technology, College of Electronic Information and Optical Engineering, Nankai University, Tianjin 300350, China; liuhaiyuan@nankai.edu.cn; 3 College of Electronic and Information Engineering, Tianjin Polytechnic University, Tianjin 300387, China; doctorxw@126.com

**Keywords:** multiple input multiple output (MIMO), decision feedback equalization (DFE), sparse representation, hybrid *l*_1_/*l*_2_ norm minimization, orthogonal matching pursuit (OMP)

## Abstract

In this paper, a novel scheme using hybrid *l*_1_/*l*_2_ norm minimization and the orthogonal matching pursuit (OMP) algorithm is proposed to design the sparse finite impulse response (FIR) decision feedback equalizers (DFE) in multiple input multiple output (MIMO) systems. To reduce the number of nonzero taps for the FIR DFE while ensuring its design accuracy, the problem of designing a sparse FIR DFE is transformed into an *l*_0_ norm minimization problem, and then the proposed scheme is used to obtain the sparse solution. In the proposed scheme, a sequence of minimum weighted *l*_2_ norm problems is solved using the OMP algorithm. The nonzero taps positions can be corrected with the different weights in the diagonal weighting matrix which is computed through the hybrid *l*_1_/*l*_2_ norm minimization. The simulation results verify that the sparse FIR MIMO DFEs designed by the proposed scheme get a significant reduction in the number of nonzero taps with a small performance loss compared to the non-sparse optimum DFE under the minimum mean square error (MMSE) criterion. In addition, the proposed scheme provides better design accuracy than the OMP algorithm with the same sparsity level.

## 1. Introduction

In high-speed wireless communication systems, the multipath effect in channels will cause severe intersymbol interference (ISI). The orthogonal frequency division multiplexing (OFDM) system [1,2,3], with the presence of the orthogonal subcarriers and the cyclic prefix, has a strong ability to combat the ISI and generally requires simple equalization. However, compared with OFDM, the single-carrier communication has the advantage of low peak-to-average power ratio of the transmitted signal and insensitivity to carrier frequency offsets [4]. Therefore, the single-carrier communication is widely applied in many communication systems, such as in underwater acoustic communication [5,6,7] and vehicular communication systems [8]. One of the widely used methods to reduce the ISI in single-carrier systems is employing the finite impulse response (FIR) decision feedback equalizers (DFE) with a large number of taps to filter the received signals. However, the design complexity of FIR DFEs grows proportional to the square of the number of nonzero taps. Especially in multiple input multiple output (MIMO) systems, the design complexity can be much higher because the number of DFE taps grows proportional to the number of multiple antennas [9]. Therefore, to reduce the design complexity, it is necessary to design the FIR DFE with only a few nonzero taps (i.e., sparse FIR DFE). In [10], the number of nonzero taps is reduced by choosing only a few significant taps of the minimum mean square error (MMSE) equalizer which is not sparse in general. Nevertheless, the non-sparse MMSE solution must be calculated first which increases the design complexity. Therefore, how to determine the positions and values of nonzero taps directly is one of the main difficulties to design the sparse FIR DFE.

In recent years, some new ways of designing sparse FIR DFE have arisen with the research and application of sparse representation and compressed sensing theory [11,12,13,14,15,16]. Among them, the compressed sensing framework for sparse signal processing is proposed in [11]. A greedy search method to determine the nonzero taps positions of a single input single output (SISO) system is presented in [12]. The *l*_1_ norm minimization method to design sparse filters is proposed in [13], but the modeling without wireless channel is quite different from the sparse equalizer design. In [14], a sparse adaptive equalizer design approach using the stochastic gradient pursuit algorithm based on compressed sensing is proposed, but the channel impulse response in the approach is assumed to be sparse. The new design frameworks for sparse FIR MIMO equalizers based on the orthogonal matching pursuit algorithm (OMP) and sparse approximation of a vector using different dictionaries are exploited in [15,16], respectively. They achieve better performance than the method in [10] with the same number of nonzero taps. Furthermore, for the pursuit of both sparse promotion and improved accuracy, the sparse signals reconstruction algorithms inspired by *l*_1_ and weighted *l*_1_ regularization schemes are proposed in [17,18]; the joint smoothed *l*_0_ norm algorithm for direction-of-arrival estimation in MIMO radar is proposed in [19].

We aim to reduce the number of nonzero taps for the FIR DFE while ensuring its design accuracy by applying the OMP algorithm to hybrid *l*_1_/*l*_2_ norm minimization. The OMP algorithm is a popular greedy algorithm that determines a nonzero tap position for each iteration [20]. However, there is no correction for the selected nonzero taps positions in the previous iterations, which will reduce the design accuracy for the sparse FIR DFE. The hybrid *l*_1_/*l*_2_ norm minimization is proposed in [21] to make the solution for data fitting less sensitive to large residuals and more robust, and it has been applied in many fields such as geophysics, image processing and machine learning. Within this context, we propose a scheme using hybrid *l*_1_/*l*_2_ norm minimization and OMP algorithm. In the proposed scheme, a minimum weighted *l*_2_ norm problems is solved using the OMP algorithm in each iteration, and the nonzero taps positions can be corrected with the iteratively updated weights in the diagonal weighting matrix, then the weighting matrix is updated through the hybrid *l*_1_/*l*_2_ norm minimization.

In this paper, we transform the problem of designing a sparse FIR MIMO DFE into an *l*_0_ norm minimization problem, then use the proposed scheme to solve the *l*_0_ norm minimization problem and obtain the sparse solution. It is verified through simulations that, when compared with the non-sparse optimum MMSE DFE, the sparse FIR MIMO DFEs designed by the proposed scheme obtain a significant reduction in the number of nonzero taps with a small performance loss. In addition, the proposed scheme provides better design accuracy than the OMP algorithm with the same nonzero taps percentage.

## 2. System Model

Consider a baseband equivalent discrete signal model in the MIMO systems with *n_i_* transmit (input) antennas and *n_o_* receive (output) antennas. Assuming that the channel is a linear time-invariant Rayleigh fading channel with an oversampling factor of *l*, the output of the MIMO channel at time *k*, i.e., the received samples from the *n_o_* receive antennas which are grouped into a *ln_o_* × 1 vector can be formed as:

(1) $$y_{k}=\left[\begin{array}{c} y_{k}^{\left(1\right)}\\ y_{k}^{\left(2\right)}\\ \vdots \\ y_{k}^{\left(n_{o}\right)} \end{array}\right]=\left[\begin{array}{cccc} H_{0} & H_{1} & \cdots & H_{\nu } \end{array}\right]\left[\begin{array}{c} x_{k}\\ x_{k-1}\\ \vdots \\ x_{k-\nu } \end{array}\right]+\left[\begin{array}{c} n_{k}^{\left(1\right)}\\ n_{k}^{\left(2\right)}\\ \vdots \\ n_{k}^{\left(n_{o}\right)} \end{array}\right],$$

where $y_{k}^{\left(j\right)}={\left[y_{k}^{\left(j\right)}(1),y_{k}^{\left(j\right)}(2),\cdots ,y_{k}^{\left(j\right)}(l)\right]}^{T}(1\leq j\leq n_{o})$ is the *l* × 1 output vector of the *j*-th receive antennas, $H_{m}(0\leq m\leq \nu )$ is the *ln_o_* × *n_i_ m-*th MIMO channel matrix, $x_{k-m}$ is the input vector of the transmit antennas at time (k−m). The parameter *ν* is the maximum length of all the subchannel impulse response. $n_{k}^{\left(j\right)}$ is an additive white Gaussian noise vector of the *j-*th receive antennas with mean 0 and variance *σ*^2^. Considering a block of *N_f_* received vector $y_{k}$, (1) can be rewritten as [9]

(2) $$y_{k:k-N_{f}+1}=Hx_{k:k-N_{f}-\nu +1}+n_{k:k-N_{f}+1},$$

where

(3) $$H=\left[\begin{array}{cc} \begin{array}{cccc} H_{0} & H_{1} & \cdots & H_{\nu }\\ 0 & H_{0} & H_{1} & \cdots \\ \vdots & \ddots & \ddots & \ddots \\ 0 & \cdots & 0 & 0 \end{array} & \begin{array}{cccc} 0 & 0 & \cdots & 0\\ H_{\nu } & 0 & \cdots & 0\\ \ddots & \ddots & \ddots & \vdots \\ H_{0} & H_{1} & \cdots & H_{\nu } \end{array} \end{array}\right].$$

Assuming that the transmit signals are independent with the average power $\varepsilon_{x}$, the input autocorrelation matrix can be expressed as

(4) $$R_{xx}≜E\left[x_{k:k-N_{f}-\nu +1}x_{k:k-N_{f}-\nu +1}^{H}\right]=\varepsilon_{x}I_{n_{i}(N_{f}+\nu )},$$

where ***I*** is the identity matrix, and the noise autocorrelation matrix can be expressed as

(5) $$R_{nn}≜E\left[n_{k:k-N_{f}+1}n_{k:k-N_{f}+1}^{H}\right]=\sigma^{2}I_{n_{0}N_{f}}.$$

Furthermore, the output-input crosscorrelation and the output autocorrelation matrices are as follows

(6) $$R_{yx}≜E\left[y_{k:k-N_{f}+1}x_{k:k-N_{f}-\nu +1}^{H}\right]=HR_{xx},$$

(7) $$R_{yy}≜E\left[y_{k:k-N_{f}+1}y_{k:k-N_{f}+1}^{H}\right]=HR_{xx}H^{H}+R_{nn}.$$

## 3. Design of Sparse FIR DFE Using Hybrid *l*_1_/*l*_2_ Norm Minimization and OMP Algorithm

In the FIR MIMO DFE model, in order to eliminate ISI and inter-antenna interference, then recover the input signal of the *i*-th transmit antenna, the feedforward filter $w_{i}$ with *ln_o_N_f_* taps processes the received signals $y_{k}$, and the feedback filter $b_{i}$ with *n_i_N_b_* taps convolves with the previous decision signals, then the result combined both together is applied to the decision device to obtain current decision signal. If the previous decision signals before time *k* are correct, the DFE error sample at time *k* for the *i*-th input antenna is given by

(8) $$e_{k}^{\left(i\right)}=x_{k-\Delta }^{\left(i\right)}-\underset{≜{\tilde{w}}_{i}^{H}}{\underset{︸}{\left[w_{i}^{H}\;-b_{i}^{H}\right]}}\underset{≜\tilde{y}}{\underset{︸}{\left[\begin{array}{c} y_{k:k-N_{f}+1}\\ x_{k-△-1:k-△-N_{b}} \end{array}\right]}},$$

where $x_{k-\Delta }^{\left(i\right)}$ is the transmit signal at time (k−Δ) by the *i-*th input antenna, and Δ is an integer representing the decision delay, usually $0\leq \Delta \leq N_{f}+\nu -1$. Using $N=ln_{o}N_{f}+n_{i}N_{b}$ to represent the number of DFE taps for simplicity, so ${\tilde{w}}_{i}$ is an N×1 vector. Then the equalizer mean square error (MSE) for the *i*-th input antenna is expressed as

(9) $$\begin{array}{ll} \xi_{i} & ≜E\left[{\left|e_{k}^{\left(i\right)}\right|}^{2}\right]=E\left[{\left|x_{k-\Delta }^{\left(i\right)}-{\tilde{w}}_{i}^{H}\tilde{y}\right|}^{2}\right]\\ & =\varepsilon_{x}-{\tilde{w}}_{i}^{H}{\tilde{r}}_{\Delta ,i}-{\tilde{r}}_{\Delta ,i}^{H}{\tilde{w}}_{i}+{\tilde{w}}_{i}^{H}R_{\tilde{y}\tilde{y}}{\tilde{w}}_{i} \end{array},$$

where $R_{\tilde{y}\tilde{y}}≜E\left[\tilde{y}{\tilde{y}}^{H}\right]$, ${\tilde{r}}_{\Delta ,i}=R_{\tilde{y}x}1_{\Delta ,i}$, $R_{\tilde{y}x}≜E\left[\tilde{y}x_{k:k-N_{f}-\nu +1}^{H}\right]$ and $1_{\Delta ,i}$ is the ($n_{i}\Delta +i$)-th column of $I_{n_{i}(N_{f}+\nu )}$. Performing the Cholesky decomposition $R_{\tilde{y}\tilde{y}}=\tilde{L}{\tilde{L}}^{H}$, where $\tilde{L}$ is an N×N lower triangular matrix, (9) can be rewritten as

(10) $$\xi_{i}=\underset{≜\xi_{\min,i}}{\underset{︸}{\varepsilon_{x}-{\tilde{r}}_{\Delta ,i}^{H}{\tilde{L}}^{-H}{\tilde{L}}^{-1}{\tilde{r}}_{\Delta ,i}}}+\underset{≜\xi_{\mathrm{excess},i}}{\underset{︸}{{\left\|{\tilde{L}}^{H}{\tilde{w}}_{i}-{\tilde{L}}^{-1}{\tilde{r}}_{\Delta ,i}\right\|}_{2}^{2}}}.$$

From (10) it shows that the different choice of ${\tilde{w}}_{i}$ can only change $\xi_{\mathrm{excess},i}$, and $\xi_{\min,i}$ remains unchanged. Obviously, $\xi_{\mathrm{excess},i}\geq 0$. Therefore, when $\xi_{\mathrm{excess},i}=0$, ${\tilde{w}}_{i}$ achieves the optimum solution under the MMSE criterion as

(11) $${\tilde{w}}_{\mathrm{opt},i}={\tilde{L}}^{-H}{\tilde{L}}^{-1}{\tilde{r}}_{\Delta ,i}=R_{\tilde{y}\tilde{y}}^{-1}{\tilde{r}}_{\Delta ,i}.$$

${\tilde{w}}_{\mathrm{opt},i}$ is usually non-sparse with too much complexity to compute directly. Therefore, in order to reduce the computational complexity, considering to reduce the number of the DFE nonzero taps at the cost of a small increase in the equalizer MSE, i.e., finding the sparse solution ${\tilde{w}}_{s,i}$ for ${\tilde{w}}_{i}$ when $\xi_{\mathrm{excess},i}\leq \theta \;(\theta >0)$, the sparse FIR MIMO DFE design problem can be transformed into the *l*_0_ norm minimization problem as follows [15]

(12) $${\tilde{w}}_{s,i}=\underset{{\tilde{w}}_{i}}{\arg\min}{\left\|{\tilde{w}}_{i}\right\|}_{0}\;\mathrm{subject}\;\mathrm{to}\;{\left\|{\tilde{L}}^{H}{\tilde{w}}_{i}-{\tilde{L}}^{-1}{\tilde{r}}_{\Delta ,i}\right\|}_{2}^{2}\leq \theta .$$

In general, finding the optimal solution of the above *l*_0_ norm minimization problem is NP-hard. One of the approaches to computing the suboptimal solution for the approximation of (12) is greedy algorithms. To design the sparse solution for the FIR MIMO DFE, in this paper, we propose the scheme based on the OMP algorithm which is a widely-used greedy algorithm.

The OMP algorithm selects an atom from the matrix ${\tilde{L}}^{H}$ (i.e., the column vector of the matrix ${\tilde{L}}^{H}$) that is most correlated with the current residual, then solves a constraint least squares problem to obtain all the selected atoms coefficients and updates the residual in each iteration. There is no correction for the selected nonzero taps positions in OMP algorithm, which will reduce the design accuracy for the sparse FIR DFE. To solve the problem, in this paper, we propose the scheme using hybrid *l*_1_/*l*_2_ norm minimization and OMP algorithm.

According to the hybrid *l*_1_/*l*_2_ norm minimization, at the (k+1)-th iteration, the proposed scheme will reweight the residual vector $r={\tilde{L}}^{H}{\tilde{w}}_{i}-{\tilde{L}}^{-1}{\tilde{r}}_{\Delta ,i}$ with an N×N iteratively updated diagonal weighting matrix $Q^{\left(k\right)}$, and minimize the weighted *l*_2_ norm

(13) $$J({\tilde{w}}_{i})={\left\|Q^{\left(k\right)}{\tilde{L}}^{H}{\tilde{w}}_{i}-Q^{\left(k\right)}{\tilde{L}}^{-1}{\tilde{r}}_{\Delta ,i}\right\|}_{2}^{2}.$$

Then solve the above problem using the OMP algorithm to find a sparse solution. $Q^{\left(k\right)}$ is updated from the current residual through the hybrid *l*_1_/*l*_2_ norm minimization. The diagonal entries of $Q^{\left(k\right)}$ are as follows

(14) $$q_{nn}^{\left(k\right)}=\frac{1}{{\left(1+{\left(r_{n}^{\left(k\right)}/\mu \right)}^{2}\right)}^{1/4}},\;1\leq n\leq N,$$

where $r_{n}^{\left(k\right)}$ is the *n*-th entry of the current residual $r^{\left(k\right)}$ and μ is determined by the sparse solution. Substituting (14) into (13), minimizing (13) is equivalent to minimizing

(15) $$J({\tilde{w}}_{i})=\sum_{n=1}^{N}j(r_{n}^{\left(k\right)})=\sum_{i=1}^{N}\frac{{\left(r_{n}^{\left(k\right)}\right)}^{2}}{\sqrt{1+{\left(r_{n}^{\left(k\right)}/\mu \right)}^{2}}}.$$

For an arbitrary value of $r_{n}^{\left(k\right)}$, the approximation of $j(r_{n}^{\left(k\right)})$ is

(16) $$j(r_{n}^{\left(k\right)})\approx \{\begin{array}{c} {\left(r_{n}^{\left(k\right)}\right)}^{2},\;\left|r_{n}^{\left(k\right)}\right|\ll \mu \\ \mu \left|r_{n}^{\left(k\right)}\right|,\;\left|r_{n}^{\left(k\right)}\right|\gg \mu \; \end{array}$$

which suggests $J({\tilde{w}}_{i})$ behaves like an *l*_2_ norm for small residuals and like an *l*_1_ norm for large residuals. Therefore, the hybrid *l*_1_/*l*_2_ norm minimization will make the sparse solution more robust and accurate by decreasing the sensitivity of the solution to large residuals.

The procedures of the proposed scheme for finding the sparse solution of the FIR MIMO DFE are as follows:

*Initialization*: Set the initial residual $r^{\left(0\right)}={\tilde{L}}^{-1}{\tilde{r}}_{\Delta ,i}$, define the index set Ω={1,2,⋯,N}, and initialize the diagonal weighting matrix $Q^{\left(0\right)}=\mathrm{diag}(1,1,\cdots ,1)$ for the first iteration.

*Main iteration*: Set *k* = 1 and perform the following steps:

*Step 1*: Reweight the residual vector $r={\tilde{L}}^{H}{\tilde{w}}_{i}-{\tilde{L}}^{-1}{\tilde{r}}_{\Delta ,i}$ and formulate the problem

(17) $$\min{\left\|Q^{\left(k-1\right)}{\tilde{L}}^{H}{\tilde{w}}_{i}-Q^{\left(k-1\right)}{\tilde{L}}^{-1}{\tilde{r}}_{\Delta ,i}\right\|}_{2}^{2}\;\mathrm{subject}\;\mathrm{to}\;{\left\|{\tilde{w}}_{i}\right\|}_{0}\leq k.$$

Set $A^{\left(k\right)}=Q^{\left(k-1\right)}{\tilde{L}}^{H}$ and normalize the column vectors ${\left\{a_{j}^{\left(k\right)}\right\}}_{j=1}^{N}$ of the matrix $A^{\left(k\right)}$ as follows

(18) $$d^{\left(k\right)}=A^{\left(k\right)}\Lambda^{\left(k\right)}=[d_{1}^{\left(k\right)},d_{2}^{\left(k\right)},\cdots ,d_{N}^{\left(k\right)}]$$

where $\Lambda^{\left(k\right)}=\mathrm{diag}\left(1/{\left\|a_{1}^{\left(k\right)}\right\|}_{2},1/{\left\|a_{2}^{\left(k\right)}\right\|}_{2},\cdots ,1/{\left\|a_{N}^{\left(k\right)}\right\|}_{2}\right)$. Set $z^{\left(k\right)}={\left(\Lambda^{\left(k\right)}\right)}^{-1}{\tilde{w}}_{i}$ and $\beta^{\left(k\right)}=Q^{\left(k-1\right)}{\tilde{L}}^{-1}{\tilde{r}}_{\Delta ,i}$, then (14) becomes

(19) $$\min{\left\|D^{\left(k\right)}z^{\left(k\right)}-\beta^{\left(k\right)}\right\|}_{2}^{2}\;\mathrm{subject}\;\mathrm{to}\;{\left\|z^{\left(k\right)}\right\|}_{0}\leq k$$

*Step 2*: Solve (16) with the OMP algorithm. Set the equalizer nonzero taps initial index set $I_{0}=\emptyset$, initialize the residual $r_{0}=\beta^{\left(k\right)}$. In the *t*-th (1≤t≤k) iteration, the process is as follows:

(1) Calculate the correlation between the residual $r_{t-1}$ and the column vector of the matrix $D^{\left(k\right)}$, and find the index $\lambda_{t}$ to the vector $d_{\lambda_{t}}^{\left(k\right)}$ which is most correlated with $r_{t-1}$, i.e., $\lambda_{t}=\arg\underset{j}{\max}\left|\langle r_{t-1},d_{j}^{\left(k\right)}\rangle \right|,j\in \Omega -I_{t-1}$;

(2) Update the nonzero taps index set $I_{t}=I_{t-1}\cup \{\lambda_{t}\}$, and then update the set of column vectors $\Phi_{t}^{\left(k\right)}=[d_{\lambda_{1}}^{\left(k\right)},d_{\lambda_{2}}^{\left(k\right)},\cdots ,d_{\lambda_{t}}^{\left(k\right)}]$ corresponding to $I_{t}$;

(3) Compute the sparse solution ${\hat{z}}_{t}^{\left(k\right)}=\mathrm{argmin}{\left\|\Phi_{t}^{\left(k\right)}z_{t}^{\left(k\right)}-\beta^{\left(k\right)}\right\|}_{2}$, and update the residual $r_{t}=\beta^{\left(k\right)}-\Phi_{t}^{\left(k\right)}{\hat{z}}_{t}^{\left(k\right)}$;

(4) Check the value of *t*. If *t = k*, update the sparse solution ${\hat{z}}^{\left(k\right)}={\hat{z}}_{t}^{\left(k\right)}$ and residual $r^{\left(k\right)}={\left(Q^{\left(k-1\right)}\right)}^{-1}r_{t}$, then proceed to *Step 3*, else set *t* = *t* + 1 and go to (1).

*Step 3*: Check the stopping rule whether ${\left\|r^{\left(k\right)}\right\|}_{2}^{2}\leq \theta$. If met, get the sparse solution ${\tilde{w}}_{s,i}=\Lambda^{\left(k\right)}{\hat{z}}^{\left(k\right)}$ for the FIR DFE, and exit the algorithm; else update the diagonal weighting matrix $Q^{\left(k\right)}$ whose diagonal entries are determined by (14). From [22], set $\mu =\max\left(\left|{\hat{z}}^{\left(k\right)}\right|\right)/100$. Then set *k* = *k* + 1 and go to *Step 1*.

## 4. Simulation Results

In the following, the simulation results are provided to verify the performance of the proposed scheme for sparse FIR MIMO DFE design in two different channels. As a comparison, the performance of the optimum MMSE DFE and the OMP method in [15] are presented respectively.

Two different multipath Rayleigh fading channels, namely ITU (International Telecommunication Union) Vehicular A channel [23] and an exponential decay channel, are simulated. ITU Vehicular A channel has a sparse power delay profile (PDP) with 6-paths spanning about 13 symbol periods. The exponential decay channel has a non-sparse PDP as $\rho (n)=10^{-n/10},0\leq n\leq 12$ with 13-paths. The simulation system uses binary phase shift keying (BPSK) modulation. The decision delay is set to be $\Delta =N_{f}–1$, which is optimum when *N_b_* = *ν* for DFEs [24]. The small increase θ in the equalizer MSE of the sparse DFE compared with the MMSE DFE is determined by the maximum tolerable performance loss $\eta_{\max}$ as $\theta =(10^{\eta_{\max}/10}-1)\xi_{\min}$, where $\xi_{\min}$ is the MSE for the MMSE DFE. We define the signal-to-noise (SNR) as $\mathrm{SNR}≜10\mathrm{lg}(\varepsilon_{x}/\sigma^{2})$, set the oversampling factor *l* = 2 and MIMO DFEs with *n_i_* = 2 and *n_o_* = 2.

**Figure 1:**
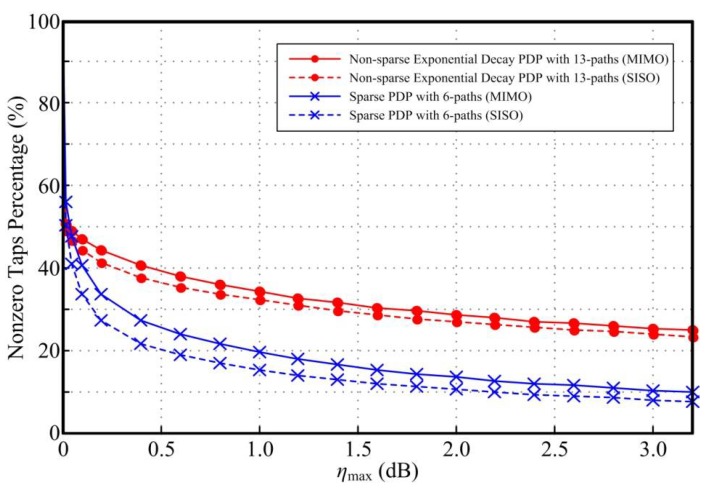
Nonzero taps percentage for the sparse SISO DFEs and MIMO DFEs versus $\eta_{\max}$ with SNR = 20 dB and *N_f_* = 30.

Figure 1 plots the nonzero taps percentage for the sparse SISO DFEs and MIMO DFEs versus the performance loss $\eta_{\max}$ using the proposed scheme respectively. It is shown that the number of nonzero taps for DFE is reduced with the increase of $\eta_{\max}$. When $\eta_{\max}$ = 0.4 dB, the nonzero taps for both the sparse SISO DFEs and MIMO DFEs are reduced by more than 70% and 60% in the two different channels respectively. Therefore, comparing the ITU Vehicular A channel with sparse PDP, the FIR DFEs needs more nonzero taps to eliminate the ISI caused by the multipath effect in the exponential decay channel with more paths. Furthermore, in MIMO systems, the DFEs needs more nonzero taps to eliminate both ISI and inter-antenna interference, as shown in Figure 1, MIMO DFEs has a larger percentage of nonzero taps when compared with SISO DFEs.

**Figure 2:**
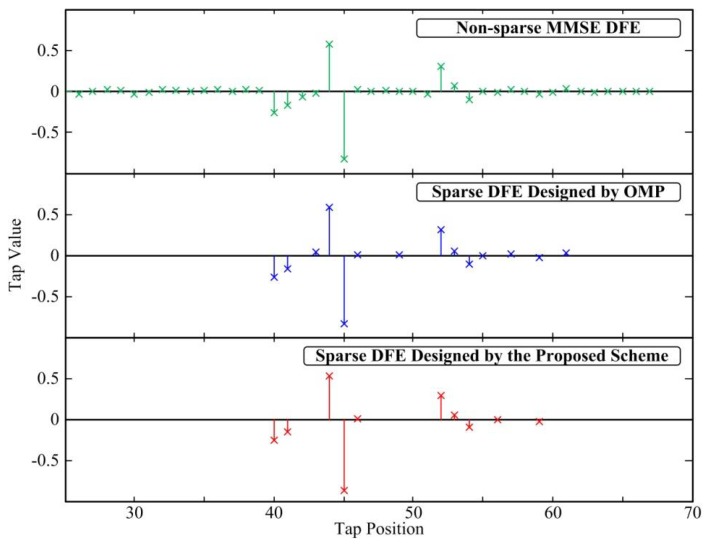
Single example of the SISO DFE by the three design method in Vehicular A channel with $\eta_{\max}$ = 0.6 dB, SNR = 20 dB and *N_f_* = 25.

Figure 2 gives a single example of the SISO DFE by the three design methods in ITU Vehicular A channel. Note that only the nonzero taps are marked, and the tap indices below 25 are insignificant to show. It is shown that there are many taps whose values are close to zero in the non-sparse MMSE DFE. Compared with the MMSE DFE, most of the taps for sparse FIR DFEs designed by the OMP method and the proposed scheme are zero, and the tap positions selected by the two methods are not identical.

**Figure 3:**
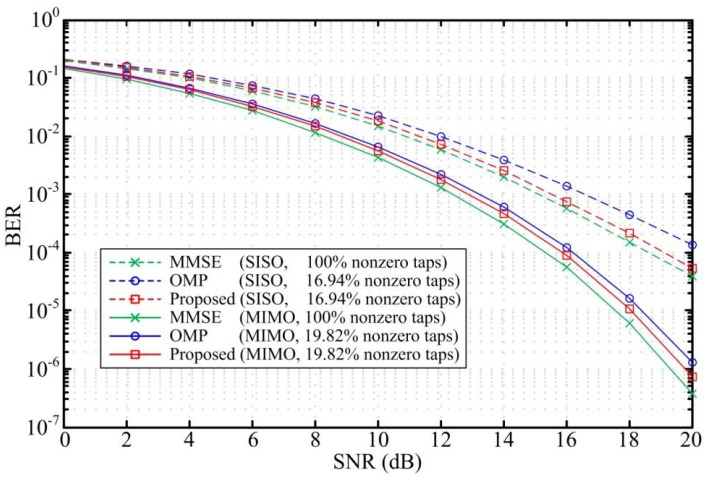
BER versus SNR for SISO DFE and MIMO DFE in the Vehicular A channel with $\eta_{\max}$ = 0.6 dB and *N_f_* = 50.

**Figure 4:**
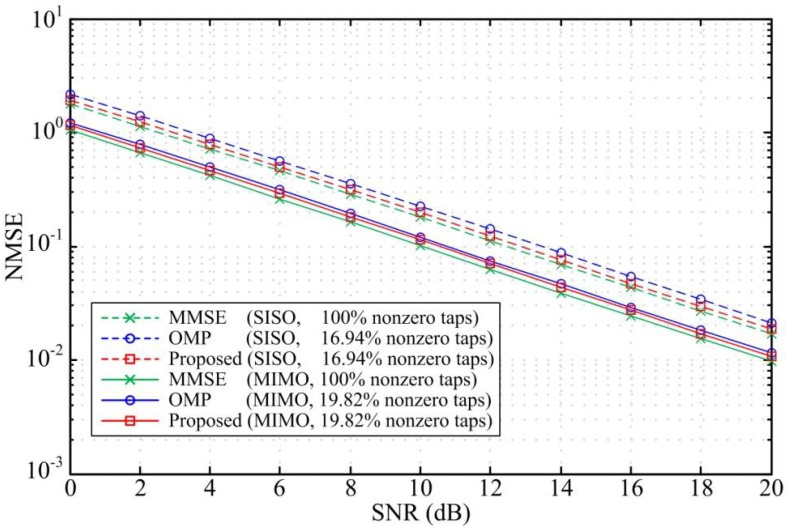
NMSE versus SNR for SISO DFE and MIMO DFE in the Vehicular A channel with $\eta_{\max}$ = 0.6 dB and *N_f_* = 50.

To verify the performance of the proposed scheme, we compare the bit error rate (BER) and the normalized mean square error (NMSE) for the three design methods. Figure 3 and Figure 4 compare the BER performance and the equalizer NMSE respectively in ITU Vehicular A channel. The sparse DFEs designed by the proposed scheme and the OMP algorithm are constrained to have the same sparsity level in the simulation. The simulation results show that the maximum SNR loss for the SISO DFEs and MIMO DFEs designed by the proposed scheme are only 0.4 dB and 0.6 dB when the number of nonzero taps is reduced by more than 83% and 80%, respectively. That is, the BER performance and the NMSE for the sparse DFEs designed by the proposed scheme are similar to the MMSE DFE performance. As shown in Figure 3, the BER performance for the sparse DFEs designed by the proposed scheme outperform the OMP algorithm with the same sparsity level. Besides, by comparing the NMSE of the two methods, it is known that the design accuracy of the proposed scheme is superior to the OMP method. Considering that the MIMO system has a certain diversity gain, the simulation results in Figure 3 show that the BER performance of the MIMO DFEs have a significant improvement than the SISO DFEs.

**Figure 5:**
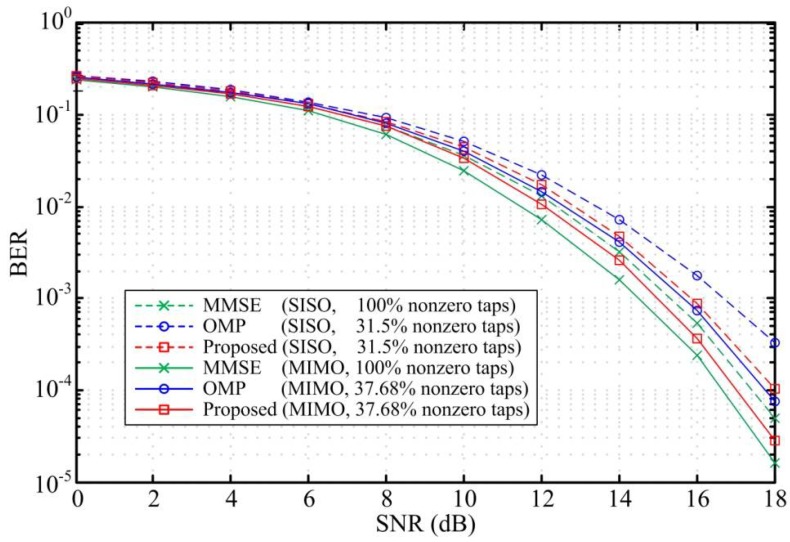
BER versus SNR for SISO DFEs and MIMO DFEs in the exponential decay channel with $\eta_{\max}$ = 0.6 dB and *N_f_* = 50.

**Figure 6:**
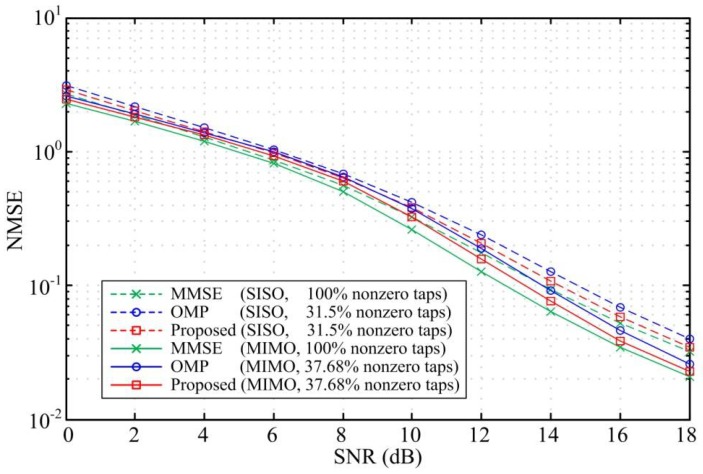
NMSE versus SNR for SISO DFEs and MIMO DFEs in the exponential decay channel with $\eta_{\max}$ = 0.6 dB and *N_f_* = 50.

To verify the performance of the proposed scheme in different channels, Figure 5 and Figure 6 show the simulation results in the exponential decay channel. Comparing with the OMP method, the results also show that the proposed scheme has better BER performance and smaller NMSE with the same sparsity level. Therefore, the proposed scheme provides excellent performance in different channel conditions.

## 5. Conclusions

In this paper, a novel scheme using hybrid *l*_1_/*l*_2_ norm minimization and OMP algorithm has been proposed to design the sparse FIR MIMO DFE. The proposed scheme solves a of minimum weighted *l*_2_ norm problem using OMP algorithm in each iteration, then the weights weighting matrix is updated through the hybrid *l*_1_/*l*_2_ norm minimization. The simulation results demonstrate that the sparse FIR MIMO DFEs designed by the proposed scheme achieve a significant reduction in the number of nonzero taps with a small performance loss compared to the non-sparse MMSE DFE. Meanwhile, the proposed scheme provides better design accuracy than the OMP algorithm with the same sparsity level.
